# An observational analysis of the impact of indoor residual spraying in Northern, Upper East, and Upper West Regions of Ghana: 2014 through 2017

**DOI:** 10.1186/s12936-020-03318-1

**Published:** 2020-07-11

**Authors:** Christelle Gogue, Joseph Wagman, Kenzie Tynuv, Andrew Saibu, Yemane Yihdego, Keziah Malm, Wahjib Mohamed, Welbeck Akplu, Titus Tagoe, Anthony Ofosu, Ignatius Williams, Samuel Asiedu, Jason Richardson, Christen Fornadel, Laurence Slutsker, Molly Robertson

**Affiliations:** 1 PATH, Washington, DC USA; 2 Abt Associates NgenIRS Project, Accra, Ghana; 3 Abt Associates Africa Indoor Residual Spraying Program, Accra, Ghana; 4 National Malaria Control Programme, Accra, Ghana; 5 Ghana Health Service, Accra, Ghana; 6 AngloGold Ashanti Malaria Control Limited, Accra, Ghana; 7 IVCC, Washington, DC USA

**Keywords:** Malaria, Indoor residual spraying, IRS, 3GIRS, Third-generation IRS, Vector control, Pirimiphosmethyl, Actellic, PM CS

## Abstract

**Background:**

Ghana has been implementing the indoor residual spraying (IRS) of insecticides since 2006, focusing operations in the north. Insecticide resistance concerns prompted a switch from pyrethroids to organophosphates, beginning gradually in 2011 and switching fully to the micro-encapsulated formulation of pirimiphosmethyl (PM CS), Actellic^®^ 300CS, a third-generation indoor residual spraying (3GIRS) product, by 2014. Entomological surveillance studies have shown IRS to be a highly effective malaria control tool, but epidemiological evidence is needed as well. Countrywide prevalence surveys have shown that malaria parasite prevalence in children under 5 years of age in Northern, Upper East, and Upper West Regions had declined to less than 40% in each region by 2016. Similarly, malaria deaths in children under 5 years of age have also been declining nationally since 2009. Although IRS is suspected to have contributed to this decline, stronger evidence is needed to link the IRS interventions to the epidemiological impact.

**Methods:**

To assess the epidemiological impact of Ghana’s IRS programmatic activities, a retrospective, observational analysis using routine epidemiological data was conducted to compare malaria incidence rates from IRS and non-IRS districts in Northern, Upper East, and Upper West Regions. Routine epidemiological data consisted of passive malaria case surveillance data reported in the District Health Information System 2 (DHIS2); with cases representing patients with suspected malaria who had sought care in the public health system and had received a confirmatory diagnosis with a positive malaria RDT result. Final routine data were extracted in September 2018. All districts that had received IRS were included in the analysis and compared to all non-IRS districts within the same region. In the Northern Region, only PMI districts were included in the analysis, as they had similar historical data.

**Results:**

District-level analysis from Northern Region from 2015 to 2017 of the aggregate malaria incidence reported from IRS districts relative to non-IRS comparator districts showed 39%, 26%, and 58% fewer confirmed malaria cases reported from IRS districts in 2015, 2016, and 2017, respectively. This translates to approximately 257,000 fewer cases than expected over the three years. In Upper East Region, the effect on reported malaria cases of withdrawing IRS from the region was striking; after spray operations were suspended in 2015, incidence increased an average of 485% per district (95% confidence interval: 330% to 640%) compared to 2014.

**Conclusions:**

The current observational analysis results are in line with the entomological studies in demonstrating the positive contribution of IRS with a 3GIRS product to malaria control programmes in northern Ghana and the value of using routine surveillance and implementation data to rapidly assess the impact of vector control interventions in operational settings, even in complex implementation environments.

## Background

Global efforts to fight malaria have proven tremendously successful in recent years, leading to a 32% reduction in malaria-attributable mortality and an 18% reduction in the incidence rate between 2010 and 2016 [1]. This success is evident in sub-Saharan Africa, which accounts for around 90% of all worldwide malaria cases; from 2010 to 2016, there was a 20% decrease in malaria incidence [2]. Much of this success has been attributed to the scale-up of mosquito control interventions, primarily the distribution of long-lasting insecticide-treated nets (LLINs) and to a lesser extent the use of indoor residual spraying (IRS) of insecticides to target malaria vectors. However, the downward trends in incidence and mortality stalled between 2015 and 2017 [1]. The continued success of malaria vector control and ultimately the goal of elimination are threatened by the rapid spread of resistance in mosquito populations to insecticides used for both LLINs and IRS [3]. As of 2016, insecticide resistance had been documented by 80% (61 of 76) of the countries that reported monitoring data to the World Health Organization (WHO) [4]. Particularly worrisome is resistance to pyrethroids—the most used class of insecticide for vector control. Pyrethroid resistance was first documented in 1993 and is now widespread across sub-Saharan Africa and beyond [5, 6]. The WHO has described the global fight against malaria as being at a cross roads, calling for increased funding and highlighting the need to develop, optimize, and implement new tools to combat malaria [7]. In vector control, it has been widely recognized that to maintain the effective use of IRS and LLINs for malaria prevention, there is an urgent need to develop and use new, non-pyrethroid insecticide products with improved residual efficacy [8–10]. To contribute to this need for investments in new tools to push towards elimination goals, the Next Generation IRS (NgenIRS) project introduced a co-payment mechanism for new products as part of a broad market-shaping effort for third-generation IRS (3GIRS) products. The first 3GIRS product to come to market was a micro-encapsulated formulation of pirimiphosmethyl (PM CS, Actellic^®^ 300CS Syngenta AG, Basel, Switzerland), an organophosphate insecticide. 3GIRS products are designed to be effective against pyrethroid-resistant mosquitoes and have an indoor residual efficacy of at least 6 months [11].

Ghana has a population of about 27 million [12], spread unequally over the country’s ten administrative regions, and malaria poses a major threat nationwide. In 2014, malaria caused more than 8 million outpatient cases [5, 13], 2200 deaths in health facilities, and 48.2% of all deaths in children under 5 years of age [14]. The 2014 Demographic and Health Survey reported the national malaria prevalence among children under 5 years at 26% based on microscopy and 36% based on rapid diagnostic test (RDT) results [15]. In 2016, the Malaria Indicator Survey reported the RDT prevalence in children under 5 years at 28% [16]. This reduction of prevalence from 2014 to 2016 was due in part to both universal distribution of LLINs nationwide and implementation of IRS in targeted districts, which have also contributed to the reduction in malaria incidence and mortality [17, 18].

IRS has been supported by the AngloGold Ashanti Malaria Control Programme (AGAMal) since 2006 and by the US President’s Malaria Initiative/Africa Indoor Residual Spraying Project (PMI/AIRS) since 2008. Various districts in Northern, Upper West, Upper East, Ashanti, Western, and Central Regions have been sprayed.

As the country continues to expand its use of tools, such as 3GIRS products, to attain its goal of elimination, the retrospective observational analyses here describe the impact of (1) the PMI-supported IRS campaigns implemented in Northern Region from 2015 to 2017 and (2) the AGAMal-supported IRS campaigns implemented in Upper East and Upper West Regions in the same years.

## Methods

### Study sites

The analyses focused on data collected during 2015 through 2017 from the northern savannah of Ghana, namely Northern, Upper East, and Upper West Regions, which in 2014 reported malaria prevalence by RDT of 60.6%, 22.7%, and 62.3%, respectively [14]. Although transmission occurs year-round in this area, peak transmission usually occurs from July to November [19]. The primary malaria vectors in Ghana are those belonging to the *Anopheles gambiae* species complex and *Anopheles funestus* [20]. Key components of the National Malaria Control Programme (NMCP) strategy include integrated vector control through universal coverage of LLINs, and IRS targeted to districts with greater than 40% parasite prevalence. Following a mass campaign in 2014, during which 14 million LLINs were distributed, the percentage of households owning at least one LLIN increased in both rural (78.4 to 82.4%) and urban (60.1 to 65.3%) areas from 2014 to 2016 [14, 21]. LLIN access and usage patterns were similar across the regions during the study period, as was access to malaria testing and treatment (Table 1) [14, 22]. Multiple districts in the northern regions have benefitted from IRS efforts supported by PMI/AIRS (Northern Region) and AGAMal (Upper East and Upper West Regions).

**Table 1:** Malaria prevalence by RDT and ITN coverage in the study regions

	Northern Region		Upper East Region		Upper West Region	
	2014 DHS	2016 MIS	2014 DHS	2016 MIS	2014 DHS	2016 MIS
u5 RDT positive prevalence	60.6%	39.3%	22.7%	25.8%	62.3%	27.8%
HH with at least 1 ITN	71.3%	83.7%	72.8%	93.9%	77.4%	89.7%
ITNs per HH	1.7	2.4	1.5	3.0	1.5	2.1
Used ITN last night (all)	36.0%	50.7%	32.1%	63.2%	37.6%	54.0%
Used ITN last night (u5)	43.2%	61.0%	37.4%	75.5%	54.5%	60.7%

DHS: Demographic and Health Survey; HH: household; ITN: insecticide treated net; MIS: Malaria Indicator Survey; RDT: rapid diagnostic test; u5: children under 5 years old

### Indoor residual spraying intervention

The two IRS programmes, the AGAMal programme, which began activities in 2006, and the PMI/AIRS programme, which began in 2008, have been operating in selected districts based on malaria burden and technical feasibility. The PMI/AIRS programme focuses on up to 11 districts in Northern Region and the AGAMal programme on varying select districts in Ashanti, Upper East, Upper West, Northern, Western, and Central Regions. Insecticide resistance concerns prompted a switch from pyrethroids to the organophosphate PM CS for IRS (beginning in 2011 in AGAMal districts and 2012 in PMI districts); from 2015 through 2017, both implementers sprayed PM CS. Preliminary assessments show a significant impact of IRS with PM CS in areas of high pyrethroid resistance in Northern Region, with substantial decreases in blood smear positivity rates and population prevalence following spraying [21, 23]. Between 2015 and 2017, PM CS was the only IRS active ingredient used. The analysis of entomological surveillance data from IRS and non-IRS control districts of Northern Ghana has demonstrated the positive impact of IRS on entomological indicators of malaria transmission, including vector populations [24]; showing IRS to be a highly effective malaria control tool and positioning it as a key intervention. The NMCP currently uses a targeted approach for IRS, prioritizing all houses in districts with high (greater than 40%) parasite prevalence. IRS operations were typically implemented around April and May, before and/or at the beginning of the high malaria transmission season, which also coincides with the rainy season. As noted by the WHO, timeliness of campaigns is a key factor to reap maximum benefits; that is, campaigns should be implemented in the shortest period of time just prior to the onset of the transmission season [25].

### Seasonal malaria chemoprevention intervention

During the study period, Ghana also expanded implementation of seasonal malaria chemoprevention (SMC) in children aged 3 to 59 months in areas of high seasonal transmission, including Upper East and Upper West Regions. SMC was first piloted in all districts of Upper West Region in 2015, and then expanded to cover all districts in Upper East Region in 2016. The number of monthly rounds of SMC varied; four rounds were delivered in 2015 and 2017, and two in 2016. Campaigns were planned to begin with the onset of the high-transmission season.

### Data sources, data quality, estimation of malaria incidence rates, and cases averted

To review trends in malaria incidence over time by month in Northern, Upper East, and Upper West Regions, passive malaria case surveillance data from the community and district level reported in the District Health Information Management System 2 (DHIMS2) were aggregated by district (i.e., the number of suspected malaria cases, number of suspected malaria cases tested, and number of confirmed malaria cases [positive RDT]). Ghana Health Services adopted the web-based health information management system, DHIMS2, in 2009 for the reporting and analysis needs of district health administrations and health facilities at all levels of the health management system. For these analyses, routine data were first extracted from DHIMS in November 2016, with final updates extracted in September 2018. All districts that had received IRS were included in the analysis and compared to all non-IRS districts within the same region. In the Northern Region, only PMI districts were included in the analysis, as they had similar historical data. Population-based malaria case incidence rates were calculated using district population estimates based on the Ghana Statistical Service 2010 Population and Housing Census [12], which were adjusted for estimated yearly growth. Cases represented patients with suspected malaria who had sought care in the public health system and had received a confirmatory diagnosis with a positive malaria RDT result.

Climate data included rainfall and recorded temperatures from 2014 to 2016 from four weather stations (one in Upper East Region, one in Upper West Region, and two in Northern Region). The analysis focused on routine health facility data collected from 2015 from 2017. For Upper East Region, 2014 and 2018 data are included to assess the impact of the removal of IRS on the 2015 transmission season; and the impact of the 2017 spray campaign—the six-month post spray period stretched into the early months of 2018. Data quality was strengthened noticeably beginning in 2014 and confirmed through data quality assessments.

Districts were stratified by IRS status (IRS or non-IRS), based on data compiled from PMI/AIRS end-of-spray reports and national malaria operational plans for Northern, Upper East, and Upper West Regions and AGAMal spray reports for Upper East Region. The incidence of RDT-confirmed malaria cases reported per 10,000 person-months was plotted by calendar month for each IRS and non-IRS strata.

To describe the seasonal impact of IRS, the cumulative incidence of RDT-positive malaria cases observed during the six months following the IRS campaign was calculated for the IRS districts and compared to the cumulative malaria incidence observed in the non-IRS districts during the same months. The six-month post spray window was derived from the expected average duration of indoor residual efficacy for a 3GIRS product [11]. Monthly wall bioassays in northern Ghana showed that insecticides lasted about seven months on cement and wooden surfaces (doors and windows) and about 6 months on mud surfaces [26]. Cases averted were estimated by applying non-IRS district incidence rates to the populations of the IRS districts to estimate an expected number of cases, then subtracting the observed number of cases.

### Data cleaning, review, and analysis

Datasets were organized, cleaned, transformed, and joined using Microsoft Excel 2013 with Power Query v2.41 (Microsoft Corporation, Redmond, WA, USA) and Tableau v10.0 (Tableau Software, Inc., Seattle, WA, USA). Descriptive statistics were calculated using Excel 2013 and Tableau v10.0.

### Northern Region analysis

The malaria control landscape in Northern Region from 2015 to 2017 is shown in Table 2. Five districts received IRS in 2015 and 2016, and operations expanded to include an additional two districts in 2017. From Northern Region, data included monthly reports from 11 previous and current PMI IRS districts (Table 2) of 26 districts total (approximately 2.8 million confirmed cases). From these districts, about 5800 monthly reports from 2015 to 2017 were aggregated from 160 to 200 health facilities (varying depending on the month and year). Data completeness varied from an average of 74% in 2015, to a much improved 94% by 2017, with no considerable difference across IRS and non-IRS districts. The primary analysis objective for Northern Region was to estimate the impact of the annual IRS campaigns by comparing monthly trends in confirmed malaria case incidence rates reported from health facilities in districts that received IRS with neighboring comparator districts that did not. Non-IRS districts chosen for comparison included all contiguous districts that were similar enough in ecological, epidemiological, and socioeconomic profiles to have been previously included in the PMI/AIRS programme (sprayed most recently in 2012) but were not sprayed from 2015 to 2017. Non-IRS and non-PMI districts were not included in this analysis.

**Table 2:** The malaria control landscape in Northern Region from 2015–2017

	2015				2016				2017			
	IRS			LLINs	IRS			LLINs	IRS			LLINs
	AI	Acceptance Rate	Structures sprayed		AI	Acceptance Rate	Structures sprayed		AI	Acceptance Rate	Structures sprayed	
Bunkpurugu-Yunyoo	OP	95%	50,417	Routine	OP	96%	50,742	UCC	OP	98%	53,760	Routine
East Mamprusi	OP	91%	60,283	Routine	OP	96%	63,057	UCC	OP	95%	69,562	Routine
Kumbungu	OP	93%	31,333	Routine	OP	94%	31,932	UCC	OP	98%	35,934	Routine
Mamprugu-Moagduri	OP	91%	18,478	Routine	OP	93%	18,767	UCC	OP	93%	22,371	Routine
West Mamprusi	OP	88%	45,424	Routine	OP	88%	46,785	UCC	OP	91%	55,429	Routine
Gushiegu	None	–	–	Routine	None	–	–	UCC	OP	90%	48,843	Routine
Karaga	None	–	–	Routine	None	–	–	UCC	OP	92%	36,976	Routine
Chereponi	None	–	–	Routine	None	–	–	UCC	None	–	–	Routine
Saboba	None	–	–	Routine	None	–	–	UCC	None	–	–	Routine
Savelugu-Nanton	None	–	–	Routine	None	–	–	UCC	None	–	–	Routine
Tolon	None	–	–	Routine	None	–	–	UCC	None	–	–	Routine

Active ingredient OP: organophosphate; Routine: distribution through ANC and EPI visits as well as a modified school distribution; UCC: Universal coverage campaign

### Upper East Region analysis

The malaria control landscape in Upper East Region during the study period is presented in Table 3. AGAMal was the IRS implementer in this region, and all districts were sprayed in 2014. Operations in the region were suspended in 2015 but reintroduced into three districts in 2017. Data included monthly reports from all 13 districts (approximately 3.9 million confirmed cases; Table 3) from about 350 health facilities (varying depending on the month and year). About 13,000 monthly reports from 2015 to 2017 were aggregated. Data completeness in Upper East Region remained relatively high across all districts throughout the analysis years, ranging from 80–93% from 2014 to 2017. The primary study objectives for Upper East Region during this time included (1) estimating the impact of having suspended IRS operations in all districts in the region after the 2014 campaign and (2) estimating the impact of reintroducing IRS into three of those districts in 2017, relative to the remaining non-IRS comparator districts whose status did not change. A difference-in-differences approach examining the percentage change in incidence recorded across districts from year to year was used in both instances.

**Table 3:** The malaria control landscape in Upper East Region from 2014–2017

	2014				2015		2016				2017					
	IRS			LLINs	IRS	LLINs	IRS	LLINs	SMC		IRS			LLINs	SMC	
	AI	Acceptance Rate (%)	Structures sprayed		AI		AI		Coverage	Rounds	AI	Acceptance Rate	Structures sprayed		Coverage (%)	Rounds
Builsa North	OP	91.7^a^	–	Routine	None	Routine	None	UCC	80.7^b^	2	OP	96.9%	40,791	Routine	83.5	4
Builsa South	OP	91.7	–	Routine	None	Routine	None	UCC	71.3	2	OP	95.6%	23,114	Routine	96.8	4
Kasena-Nankana West	OP	91.7	–	Routine	None	Routine	None	UCC	75.0	2	OP	95.8%	63,210	Routine	87.0	4
Bawku	OP	91.7	–	Routine	None	Routine	None	UCC	89.0	2	None	–	–	Routine	99.1	4
Bawku West	OP	91.7	–	Routine	None	Routine	None	UCC	88.4	2	None	–	–	Routine	98.3	4
Binduri	OP	91.7	–	Routine	None	Routine	None	UCC	87.5	2	None	–	–	Routine	99.3	4
Bolgatanga	OP	91.7	–	Routine	None	Routine	None	UCC	90.4	2	None	–	–	Routine	92.7	4
Bongo	OP	91.7	–	Routine	None	Routine	None	UCC	92.6	2	None	–	–	Routine	94.6	4
Garu-Tempane	OP	91.7	–	Routine	None	Routine	None	UCC	92.9	2	None	–	–	Routine	98.4	4
Kasena-Nankana	OP	91.7	–	Routine	None	Routine	None	UCC	76.0	2	None	–	–	Routine	91.8	4
Nabdam	OP	91.7	–	Routine	None	Routine	None	UCC	105.8	2	None	–	–	Routine	113.5	4
Pusiga	OP	91.7	–	Routine	None	Routine	None	UCC	90.2	2	None	–	–	Routine	100.8	4
Talensi	OP	91.7	–	Routine	None	Routine	None	UCC	84.6	2	None	–	–	Routine	91.6	4

Active ingredient OP: organophosphate; Routine: distribution through ANC and EPI visits as well as a modified school distribution; UCC: Universal coverage campaign

^a^Only aggregate data for the entire region was reported in 2014

^b^Average coverage across all rounds for the year

### Upper West Region analysis

The malaria control landscape in Upper West Region during the study period is presented in Table 4. This is the only region in the present analysis in which IRS (implemented by AGAMal) and SMC implementation were consistent, both across all districts and for the duration of the study period. From Upper West Region, data included monthly reports from all 11 districts (approximately 3.6 million confirmed cases; Table 4). A total of about 17,000 monthly reports from 2015 to 2017 from about 500 health facilities were aggregated (varying depending on the month and year). The steady investments to improve routine health facility data quality are reflected in the average data completeness varying across the region from 68% in 2015, to 85% by 2017. Monthly incidence rates were calculated as above and yearly trends were assessed.

**Table 4:** The malaria control landscape in Upper West Region from 2015–2017

	2015				2016						2017				
	IRS		SMC		IRS			LLINs			IRS			SMC	
	AI	Acceptance rate (%)	Coverage (%)	Rounds	AI	Acceptance rate (%)	Structures sprayed		Coverage (%)	Rounds	AI	Acceptance Rate	Structures Sprayed	Coverage (%)	Rounds
Jirapa	OP	87.6^a^	82.1^b^	4	OP	91.6	73,073	UCC	97.0	2	OP	87.7%	74,997	96.1	4
Lambussie-Karni	OP	87.6	94.6	4	OP	93.4	41,510	UCC	96.6	2	OP	88.3%	38,851	95.4	4
Lawra	OP	87.6	94.1	4	OP	91.0	83,303	UCC	95.2	2	OP	90.3%	89,449	97.8	4
Nandom^c^	OP	87.6	95.5	4	OP	91.0	83,303	UCC	97.4	2	*“*	*“*	*“*	96.5	4
Nadowli-Kaleo	OP	87.6	92.0	4	OP	95.8	96,895	UCC	95.1	2	OP	93.9%	86,122	94.9	4
Daffiama-Bussie-Issa^c^	OP	87.6	91.8	4	OP	95.8	96,895	UCC	95.5	2	*“*	*“*	*“*	97.4	4
Sissala East	OP	87.6	99.3	4	OP	97.5	48,623	UCC	98.5	2	OP	89.2%	52,468	95.3	4
Sissala West	OP	87.6	95.0	4	OP	93.0	50,310	UCC	99.0	2	OP	91.9%	49,753	98.4	4
Wa	OP	87.6	96.6	4	OP	92.6	96,158	UCC	98.1	2	OP	92.5%	105,285	97.1	4
Wa East	OP	87.6	96.4	4	OP	96.5	59,607	UCC	97.8	2	OP	92.6%	55,918	95.0	4
Wa West	OP	87.6	98.6	4	OP	94.2	57,061	UCC	99.1	2	OP	94.4%	54,226	96.8	4

Active ingredient OP: organophosphate; UCC: Universal coverage campaign

^a^Only aggregate data for the entire region was reported in 2014

^b^Average coverage across all rounds for the year

^c^Districts created after official redistricting in 2014: Nandom was previously part of Lawra; Daffiama-Bussie-Issa part of Nadowli-Kaleo. IRS coverage was reported using the previous district delineations

## Results

### Impact of indoor residual spraying in Northern Region

**Fig. 1:**
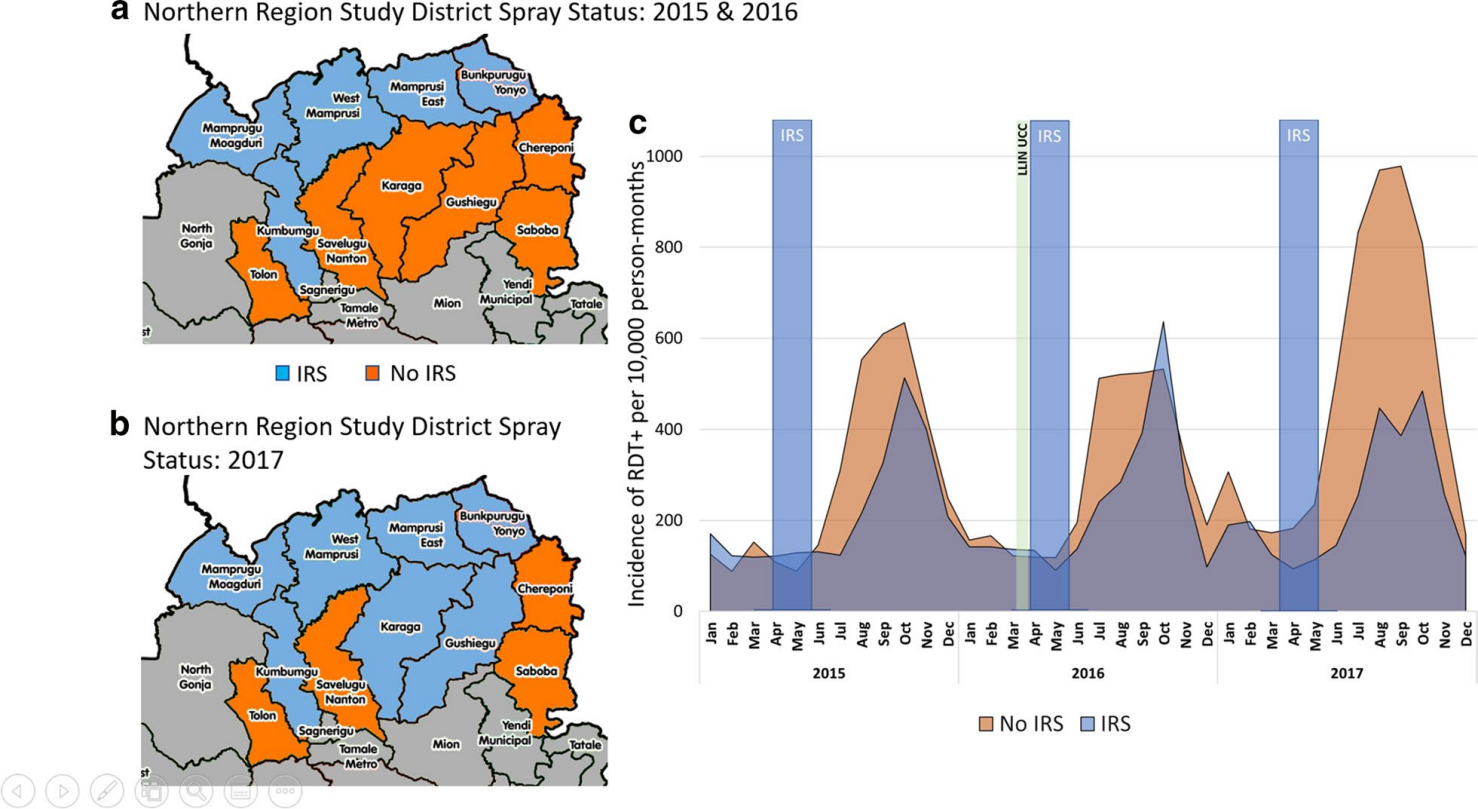
IRS Implementation in the Northern Region study districts in **a** 2015–2016 and **b** 2017; **c** Average monthly incidence of confirmed cases of malaria from DHIS2 during the study period in IRS districts (blue) and No-IRS comparator districts (orange); 39%, 26%, and 58% fewer confirmed malaria cases were reported from IRS districts in 2015, 2016, and 2017, respectively

Trends in average monthly malaria incidence rates stratified by district spray status are shown in Fig. 1. Considering the six-month window that follows each spray campaign (May to October), comparative analysis shows a much lower aggregate malaria incidence reported from IRS districts relative to non-IRS comparator districts: 39%, 26%, and 58% lower incidence of confirmed malaria cases were reported from IRS districts in 2015, 2016, and 2017, respectively, which translates to more than 257,000 fewer cases than expected over the three years (Table 5).

**Table 5:** Estimated Impact of IRS with Actellic^®^300CS in Northern Region, 2015–2017

Year	6-month cumulative incidence (May–Oct; per 10,000 person-months at risk)					
	Non-IRS Districts	IRS Districts	Cumulative Fewer Cases	Average population in IRS districts	Total population in IRS districts^a^	Cases averted
2015	2340	1438	901 (39%)	103,945	519,725	56,218
2016	2401	1779	623 (26%)	103,958	519,792	38,834
2017	4337	1830	2507 (58%)	107,738	754,168^b^	162,072
Total	9078	5047	4031 (44%)	315,641	1793,6685	257,124

^a^Out of total estimated Northern Region population—2015: 2,874,374; 2016: 2,960,606; 2017: 3,049,424

^b^In 2017 two additional districts were sprayed, hence the increase in IRS district population

### Impact of indoor residual spraying in Upper East Region

#### Elimination of indoor residual spraying in 2015

**Fig. 2:**
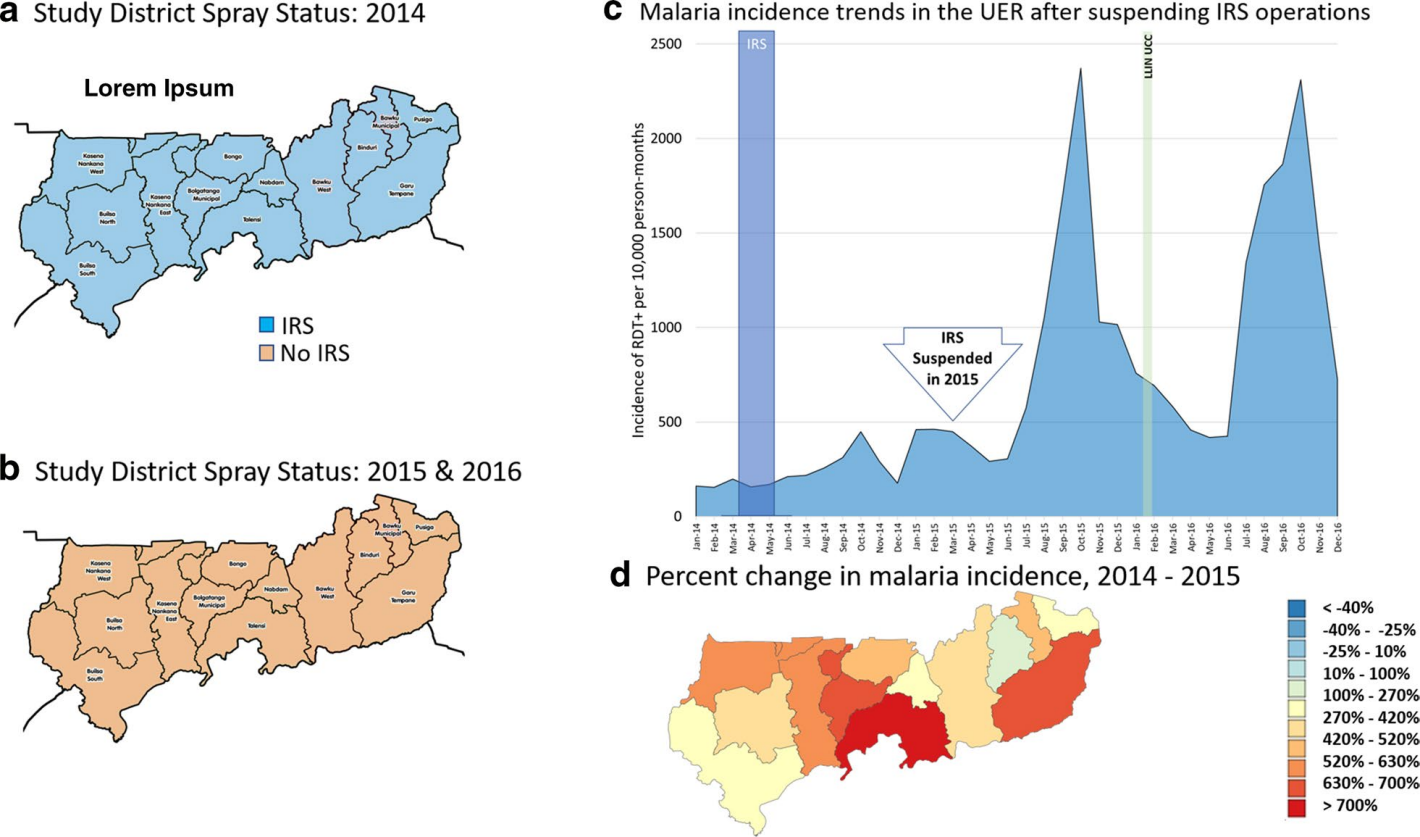
IRS Implementation in Upper East Region study districts in **a** 2014 and **b** 2015–2016; **c** Average monthly incidence of confirmed cases of malaria from DHIS2 during the study period (all study districts had the same IRS status during this timeframe). **d** The percent change in malaria incidence from 2014 to 2015 in each district, following the suspension of IRS operations

In 2014, all districts in Upper East Region were sprayed with PM CS, but IRS operations were suspended in 2015 (Fig. 2a, b). The effect on reported malaria cases of withdrawing IRS from the region was striking, as illustrated in the Fig. 2c. In 2014, the average six-month cumulative incidence (from May to October) per district was 1230 (95% confidence interval [CI_95_] 800 to 1660) confirmed cases per 10,000 person-months at risk. After spray operations were suspended in 2015, incidence during the same 6-month window increased an average of 485% per district (CI_95_ 330 to 640%), to 6115 (CI_95_ 4720 to 7500) confirmed cases per 10,000 person-months at risk. Though somewhat variable, the trend was significant (t-test on the difference of means: p < 0.00001) and consistent across all districts in the region (range: 115 to 1049% increase; Fig. 2d).

#### Reintroduction of indoor residual spraying in 2017

**Fig. 3:**
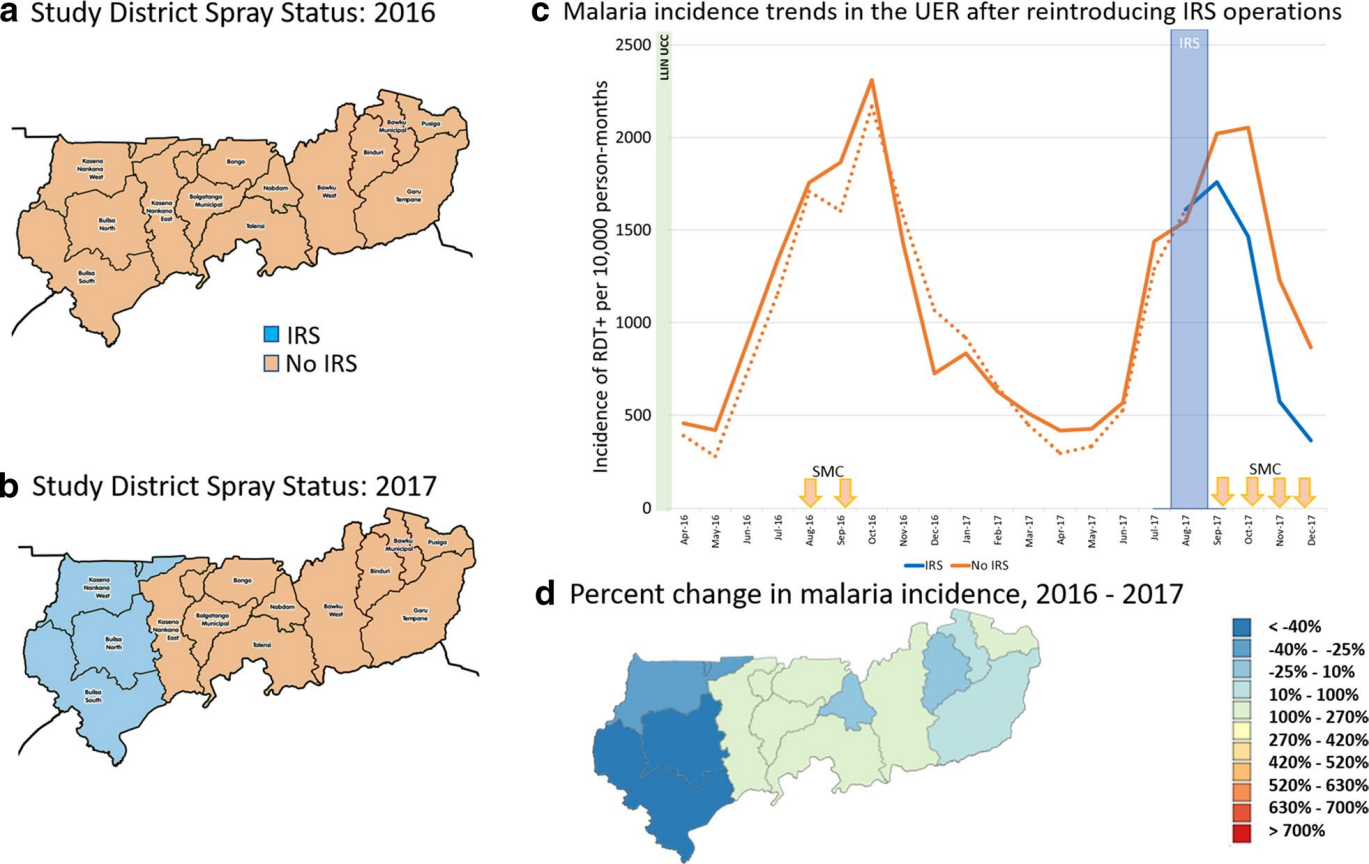
The reintroduction of IRS operations in three districts of Upper East Region in 2017. **c** Average monthly incidence of confirmed cases of malaria from DHIS2 during the study period in non-IRS (solid and dotted orange) and IRS (blue) districts; dotted lines are districts that turn blue and before IRS districts in 2017. The solid lines remained non-IRS districts in 2017. **d** The percent change in malaria incidence from 2016 to 2017 in each district, showing the substantial decrease in confirmed case incidence in the IRS districts

With available resources, IRS was reintroduced in three districts in 2017: Kassena, Builsa North, and Builsa South (Fig. 3a, b). Because of operational logistics, the spray campaign was implemented later in the transmission season than is typical, during August and September rather than April and May. The monthly incidence trends in these districts, relative to the remaining ten comparator districts of Upper East Region that received no IRS in 2016 or 2017, are shown in Fig. 3c. In the six months after the IRS campaign (September 2017 to February 2018), cumulative monthly incidence rates were 35% lower in IRS districts compared to non-IRS districts, from 7139 cases per 10,000 person-months at risk to 4626 (Fig. 3d), which translates to more than 146,000 cases averted.

A difference-in-differences analysis looking at the changes in malaria incidence from 2016 to 2017 by district is also informative: in the three districts where IRS was reintroduced in 2017, cumulative malaria incidence rates from September to February fell by an average of 42% compared to the previous year (CI_95 %_ 28 to 56%); while in the remaining districts, malaria incidence rates were essentially unchanged (a slight decrease of 5% [CI_95 %_ − 6 to 15%]; Fig. 3d), a significant difference-in-differences of 37% (CI_95 %_ 18 to 57%; p = 0.002).

### Impact of indoor residual spraying in Upper West Region

**Fig. 4:**
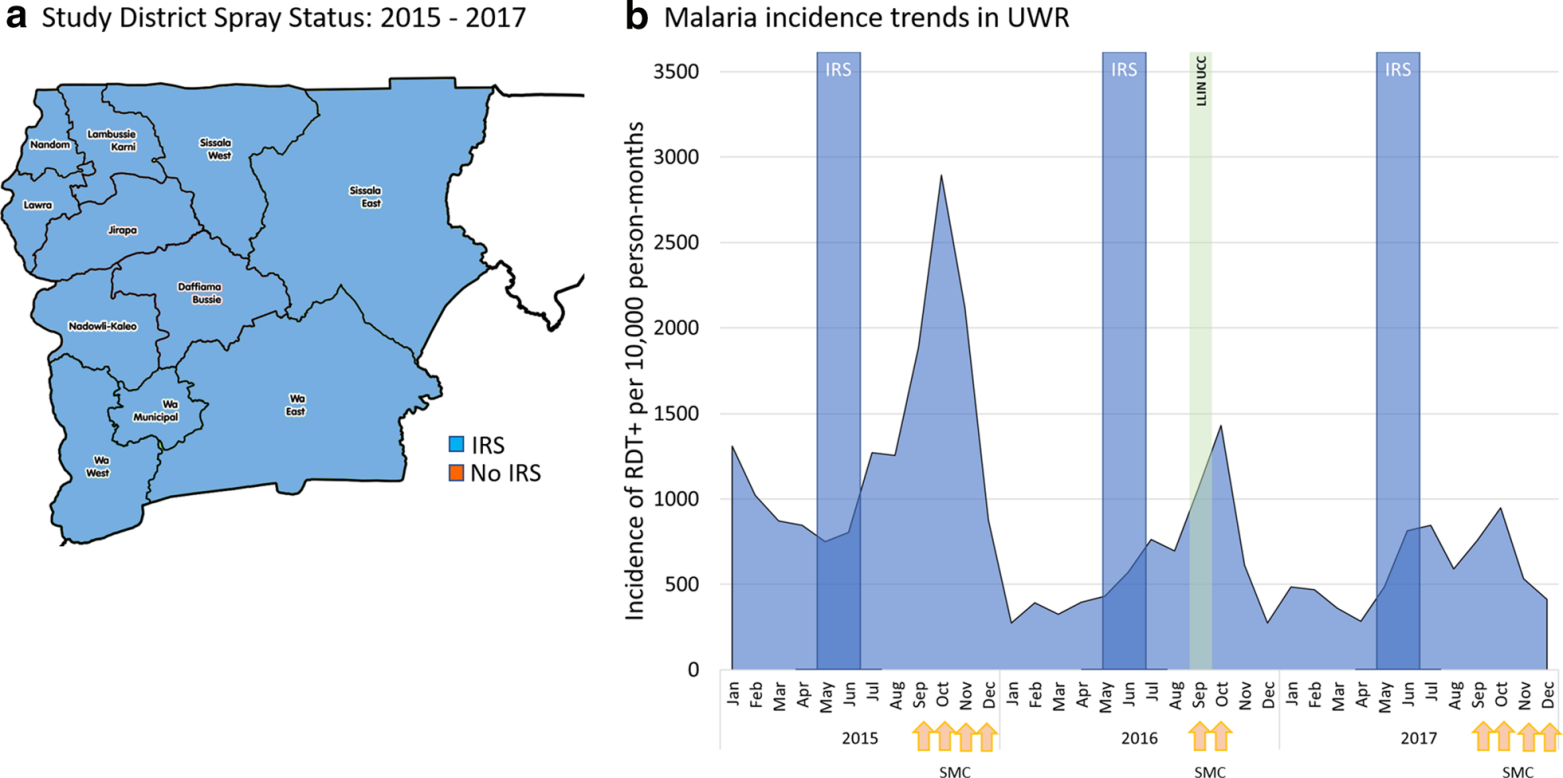
IRS status of the Districts in Upper West Region, 2015–2017. Upper West was the only region to consistently employ both IRS and SMC campaigns throughout this analysis. It was also the only region to record consistent annual drops in confirmed case incidence in the DHIS2 surveillance system across all districts (**b**)

In Upper West Region, both IRS operations and SMC implementation were consistently delivered across all districts throughout the study period (Table 4). District-level analysis of monthly reporting trends from 2015 to 2017 in this region show this to be the only study region in which incidence rates declined consistently each year (Fig. 4). Average incidence rates fell from 1500 cases per 10,000 person-months at risk in the transmission season of 2015 to 825 in 2016 and 740 in 2017, corresponding to a 44% decrease between 2015 and 2016 and a further 10% decrease between 2016 and 2017.

## Discussion

These results, from a retrospective analysis of passively reported malaria surveillance data from the northern savannah regions of Ghana, illustrate the positive contribution that IRS with 3GIRS products made to malaria control in an area of widespread pyrethroid resistance [2, 3] and universal LLIN coverage; and show the value of using quality routine surveillance information and program implementation data to efficiently assess the impact of vector control interventions in operational settings. Fortunately, implementation of both IRS and SMC allowed for good comparators within Northern and Upper East Regions, with the grouped IRS and non-IRS districts of each region being similar both ecologically and demographically. Results from Northern Region show that IRS interventions helped reduce the incidence of confirmed malaria cases reporting to public health facilities by around 40%, averting several hundred thousand cases from 2015 to 2017. In Upper East Region, the removal of IRS was followed by a resurgence of cases for 2 years, prompting the country, with the availability of additional resources, to reintroduce IRS in a few districts in 2017—a decision also clearly linked temporally and geographically with significant reductions in confirmed malaria cases reported (around 35% fewer confirmed cases after IRS). Compared to the other regions of the analysis, the incidence peaks in the non-IRS districts in 2015 and 2016 in Upper East Region stand out, supporting the idea that the suspension of the IRS campaign may have contributed to the increase in incidence. There was also an increase in prevalence between the 2014 DHS and 2016 MIS only in the Upper East Region, contrary to the Upper West and Northern regions. Similarly, analyses of the impact of suspending IRS operations in Segou Region of Mali and Apac District of Uganda, both with transmission intensity comparable to the northern savannah of Ghana, have shown a rapid resurgence of cases [27, 28].

These results are in line with the results of PMI/AIRS mosquito surveillance activities, which also consistently show an impact of IRS on reduced entomological indicators of transmission and indicate a good residual efficacy of an average of 7 months on a variety of surfaces. Data from PMI/AIRS routine entomological surveillance activities have shown lower indoor resting vector densities when comparing IRS and non-IRS sentinel sites, as well as significant reductions in entomological inoculation rates [23, 26]. The same entomological surveillance activities found that the overall sporozoite rate for both *An. gambiae* sensu lato and *An. funestus* in the IRS districts ranged from 0.74% (N = 1344) to 0.88% (N = 1812); and from 0.68% (N = 586) to 1.16% (N = 1984) in non-IRS districts. In addition, previous work in Bunkpurugu-Yunyoo, Savelugu Nanton, Tolon/Kumbungu, and Tamale Districts found that compared to IRS with a pyrethroid in 2012, IRS with PM CS in 2013 had a larger impact on reducing the indoor resting density of *An. gambiae* [26]. A substantial impact on reducing malaria parasite prevalence was observed as well: a 5% decline from 2011 (52.4%) to 2012 (47.7%), when a pyrethroid insecticide was used for IRS, compared to a 57% decline in parasite prevalence, from 47.7 to 20.6% from 2012 to 2013, following the switch in active ingredients [21], likely due to the change to the new class of insecticide to which the local vector population was susceptible. Furthermore, the combination of SMC and IRS in Upper West Region seems to have had a sustained impact on incidence reduction, where both interventions have continuously been implemented across all districts since 2015 and reductions in both incidence and prevalence have been consistently reported since 2014 [13, 14].

Although these results are positive and encouraging for IRS operations in Ghana’s northern savannah, it is important to note the limitations of this study. Ideally, for better granularity, malaria incidence would be estimated by dividing the total number of positive malaria RDT results reported from each health facility via DHIS2 by the mid-year population estimates for each health facility catchment area. However, population estimates were most reliable at the district level and reported as such in DHIS2. RDT-positive case data were therefore aggregated to the district level for analysis. In addition, although data quality assessments showed improvements in quality from 2014 onward, there would have been some data quality issues that were not documented. These analyses are heavily dependent on good data quality from health facility passive case detection. It is important to note that gaps in routine data quality remain despite the investments made to improve key surveillance components. In Ghana, these investments have specifically gone towards improved stock management, the expansion of electronic reporting, supervision, and better diagnosis. Another key limitation of using routine data for analysis includes reliance on malaria RDT results from patients who passively reported to the health system, which does not account for issues of patient access to health services that are likely to disproportionately affect more rural populations, as well as the different geographical and temporal resolutions at which various datasets were available [24]. Although seasonal spray status and monthly malaria data were available at the health facility level, standardized and reliable population data were limited to the district and/or regional levels, preventing calculations of incidence at the health facility level. It is worth noting that fever testing rates improved in the region from 2014 (79% of all fevers presented at a health facility in children under 5 years tested) to 2015 (93% of all fevers presented at a health facility in children under 5 tested), leading to improvements in appropriate diagnosis, treatment, and likely reporting, as well. It is important to note that there was not a noticeable difference in data quality attributes and care seeking between IRS and non-IRS districts. Climate data were not available at the district or health facility levels to allow the evaluation of potential differences across districts. The average monthly regional temperatures and precipitations were calculated nevertheless to identify particularly outliers within each region. The rainiest months across all 3 years and regions of analysis were July, August, and September. In addition, because malaria data prior to 2014 were not reliable, it was not possible to further the historical comparisons between IRS and non-IRS districts.

Despite the limitations, these analyses should encourage the use of routine data to evaluate malaria interventions. In the case of the northern savannah regions of Ghana, IRS with non-pyrethroid insecticides has had a positive public health impact by reducing malaria incidence rates. These new tools come at an additional financial cost to programmes. The financial resources required to sustain progress and further reduce the malaria burden remain limited. For many countries, switching to 3GIRS products can mean shifting some of the operational cost to cover the additional cost of the insecticides, therefore decreasing the geographies that will receive IRS and making decisions on where and what to prioritize. In the case of Ghana, the higher cost of the new IRS product led to suspending campaigns in the Upper East Region in 2015. Other countries have had to make similar decisions while negotiating with donors and partners for additional support. The NgenIRS project sought to reduce these costs through its market-shaping strategies; with that, malaria programs and implementation partners have been able to procure more than 4 million units of 3GIRS as prices dropped from $23.50 per unit to $15.00 per unit [11]. As part of a progressive insecticide resistance management plan, the NMCP in Ghana has since preemptively introduced a second 3GIRS product, SumiShield^®^ 50WG (Sumitomo Chemical UK PLC, London, UK), containing the neonicotinoid clothianidin, and subsequent analyses are currently underway to evaluate the public health impact of this second product. To best optimize available funding, countries and programmes must make evidence-based decisions when choosing if and how to implement malaria control interventions, including these new vector control tools.

## Conclusions

The switch from pyrethroids to organophosphates for IRS in Ghana was prompted by reported resistance to pyrethroids in local malaria vector populations. Ghana has been spraying organophosphates in Upper West Region since 2011, in Northern Region since 2012, and in Upper East Region since 2013 (with an interruption of no IRS in 2015 and 2016). The switch appears to have made a positive contribution to malaria control in the northern savannah of Ghana, as evidenced by reductions in the incidence of confirmed cases reported in the DHIS2 surveillance system consistently associated in time and space with IRS campaigns. Following two of years of stalling results in malaria reduction, the goals of elimination seemed less attainable. With a now wider toolbox of malaria interventions, countries can continue to strategize on how to maximize their resources and tailor their interventions for optimal results; utilizing observational evaluations for quick and effective decision-making.

## Data Availability

The intervention, entomology, and climate datasets used and/or analyzed during this study were consolidated from public sources and are available from the corresponding author upon reasonable request. Requests about the malaria surveillance datasets analyzed here should be directed to Anthony Ofosu, Deputy Director in charge of Monitoring and Evaluation in the Ghana Health Service.

## Abbreviations

3GIRS: Third-generation indoor residual spraying
AGAMal: AngloGold Ashanti Malaria Control Programme
CI_95_: 95% Confidence interval
DHIS2: District Health Information System 2
DHS: Demographic and Health Survey
HH: Household
IRS: Indoor residual spraying
LLIN: Long-lasting insecticide-treated net
MIS: Malaria Indicator Survey
MCP: National Malaria Control Programme
PM CS: Micro-encapsulated formulation of pirimiphosmethyl
PMI/AIRS: US President’s Malaria Initiative/Africa Indoor Residual Spraying
RDT: Rapid diagnostic test
SMC: Seasonal malaria chemoprevention
u5: Under 5 years of age
